# *N*-(4-Chloro­phenyl­sulfon­yl)-2-methyl­propanamide

**DOI:** 10.1107/S1600536811031394

**Published:** 2011-08-06

**Authors:** P. G. Nirmala, Sabine Foro, B. Thimme Gowda

**Affiliations:** a Department of Chemistry, Mangalore University, Mangalagangotri 574 199, Mangalore, India; b Institute of Materials Science, Darmstadt University of Technology, Petersenstrasse 23, D-64287 Darmstadt, Germany

## Abstract

In the crystal structure of the title compound, C_10_H_12_ClNO_3_S, the N—C bond in the C—SO_2_—NH—C segment has a *gauche* torsion with respect to the S=O bonds. The mol­ecule is twisted at the S atom with a C—S—N—C torsion angle of −62.3 (3)°. The benzene ring and the SO_2_—NH—CO—C segment form a dihedral angle of 89.3 (1)°. In the crystal, mol­ecules are linked by pairs of N—H⋯O hydrogen bonds into inversion dimers.

## Related literature

For the sulfanilamide moiety in sulfonamide drugs, see: Maren (1976 ▶). For its ability to form hydrogen bonds in the solid state, see: Yang & Guillory (1972 ▶). For hydrogen-bonding modes of sulfonamides, see: Adsmond & Grant (2001 ▶). For our studies on the effects of substituents on the structures and other aspects of *N*-(ar­yl)-amides, see: Arjunan *et al.* (2004 ▶), on *N*-(ar­yl)-methane­sulfonamides, see: Gowda *et al.* (2007 ▶), on *N*-(ar­yl)-aryl­sulfonamides, see: Gowda *et al.* (2003 ▶) and on *N*-(aryl­sulfon­yl)-amides, see: Gowda *et al.* (2008 ▶); Shakuntala *et al.* (2011 ▶).
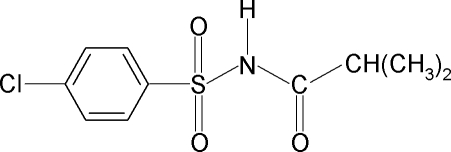


## Experimental

### 

#### Crystal data

C_10_H_12_ClNO_3_S
*M* *_r_* = 261.72
Triclinic, 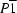

*a* = 6.207 (1) Å
*b* = 10.395 (3) Å
*c* = 10.497 (3) Å
α = 70.150 (2)°
β = 79.160 (2)°
γ = 86.010 (2)°
*V* = 625.7 (3) Å^3^
*Z* = 2
Mo *K*α radiation
μ = 0.46 mm^−1^
*T* = 293 K
0.46 × 0.20 × 0.08 mm

#### Data collection

Oxford Diffraction Xcalibur diffractometer with a Sapphire CCD detector
Absorption correction: multi-scan *CrysAlis RED* (Oxford Diffraction, 2009 ▶) *T*_min_ = 0.815, *T*_max_ = 0.964
3846 measured reflections
2476 independent reflections
1842 reflections with *I* > 2σ(*I*)
*R*_int_ = 0.014

#### Refinement

*R*[*F* ^2^ > 2σ(*F* ^2^)] = 0.060
*wR*(*F* ^2^) = 0.126
*S* = 1.19
2476 reflections
148 parameters
1 restraint
H atoms treated by a mixture of independent and constrained refinement
Δρ_max_ = 0.28 e Å^−3^
Δρ_min_ = −0.31 e Å^−3^

### 

Data collection: *CrysAlis CCD* (Oxford Diffraction, 2009 ▶); cell refinement: *CrysAlis RED* (Oxford Diffraction, 2009 ▶); data reduction: *CrysAlis RED*; program(s) used to solve structure: *SHELXS97* (Sheldrick, 2008 ▶); program(s) used to refine structure: *SHELXL97* (Sheldrick, 2008 ▶); molecular graphics: *PLATON* (Spek, 2009 ▶); software used to prepare material for publication: *SHELXL97*.

## Supplementary Material

Crystal structure: contains datablock(s) I, global. DOI: 10.1107/S1600536811031394/nc2243sup1.cif

Structure factors: contains datablock(s) I. DOI: 10.1107/S1600536811031394/nc2243Isup2.hkl

Supplementary material file. DOI: 10.1107/S1600536811031394/nc2243Isup3.cml

Additional supplementary materials:  crystallographic information; 3D view; checkCIF report

## Figures and Tables

**Table 1:** Hydrogen-bond geometry (Å, °)

*D*—H⋯*A*	*D*—H	H⋯*A*	*D*⋯*A*	*D*—H⋯*A*
N1—H1*N*⋯O1^i^	0.84 (2)	2.08 (2)	2.912 (4)	169 (3)

Symmetry code: (i) 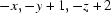.

**Fig. 1.**
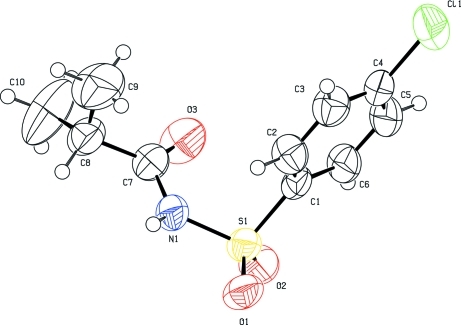
Molecular structure of the title compound, showing the atom-labelling scheme. Displacement ellipsoids are drawn at the 50% probability level.

**Fig. 2.**
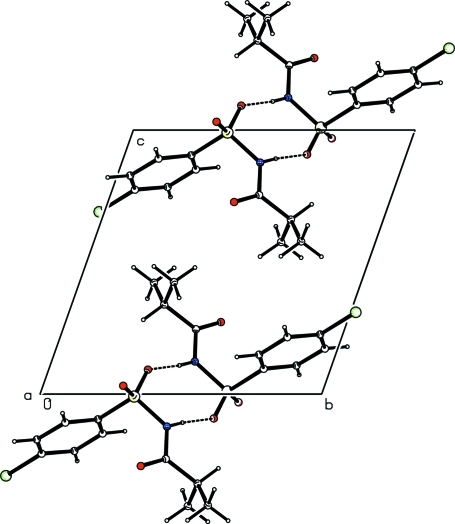
Crystal structure of the title compound showing the dimers. Hydrogen bonds are shown as dashed lines.

## supplementary crystallographic information

### Comment

The molecular structures of sulfonamide drugs contain the sulfanilamide moiety (Maren, 1976). The propensity for hydrogen bonding in the solid state, due to the presence of various hydrogen bond donors and acceptors gives rise to polymorphism (Yang & Guillory, 1972). The hydrogen bonding preferences of sulfonamides has also been studied (Adsmond & Grant, 2001). The nature and position of substituents play a significant role on the crystal structures of this class of compounds. As part of our work on the effects of substituents on the structures and other aspects of *N*-(aryl)-amides (Arjunan *et al.*, 2004), *N*-(aryl)-methanesulfonamides (Gowda *et al.*, 2007), *N*-(aryl)-arylsulfonamides (Gowda *et al.*, 2003) and *N*-(arylsulfonyl)-acetamides (Gowda *et al.*, 2008, Shakuntala *et al.*, 2011), in the present work, the crystal structure of *N*-(4-chlorophenylsulfonyl)-2,2-dimethylacetamide (I) has been determined. The N—C bond in the C—SO_2_—NH—C segment has *gauche* torsion with respect to the S═O bonds. The molecule is twisted at the *S*-atom with a C—S—N—C torsion angle of -62.3 (3)°, compared to the values of -72.5 (2)° in *N*-(4-chlorophenylsulfonyl)-2,2-dichloroacetamide (II) (Gowda *et al.*, 2008) and *N*-(2-chlorophenylsulfonyl)- 2,2-dimethylacetamide (III)(Shakuntala *et al.*, 2011).

Further, the dihedral angle between the benzene ring and the SO_2_—NH—CO—C segment in (I) is 89.3 (1)°, compared to the values of 79.7 (1)° in (II) and 87.4 (1)° in (III).

In the crystal structure, the moleucles are connected into centrosymmetrically dimers by intermolecular N–H···O hydrogen bonding (Table 1). Part of the crystal structure is shown in Fig. 2.

### Experimental

The title compound was prepared by refluxing 4-chlorobenzenesulfonamide (0.10 mole) with an excess of 2,2-dimethylacetyl chloride (0.20 mole) for about an hour on a water bath. The reaction mixture was cooled and poured into ice cold water. The resulting solid was separated, washed thoroughly with water and dissolved in warm dilute sodium hydrogen carbonate solution. The title compound was reprecipitated by acidifying the filtered solution with glacial acetic acid. It was filtered, dried and recrystallized from ethanol. The purity of the compound was checked by determining its melting point. It was further characterized by recording its infrared spectra.

Plate like colorless single crystals of the title compound used in X-ray diffraction studies were obtained from a slow evaporation of an ethanolic solution of the compound.

### Refinement

The H atom of the NH group was located in a difference map and later restrained to the distance N—H = 0.86 (2) Å The other H atoms were positioned with idealized geometry using a riding model with the aromatic C—H = 0.93 Å, methyl C—H = 0.96Å and methyne C—H = 0.98 Å.

All H atoms were refined with isotropic displacement parameters. The *U*_iso_(H) values were set at 1.2*U*_eq_(C-aromatic, N) and 1.5*U*_eq_(*C*-methyl).

### Crystal data

**Table:**

C_10_H_12_ClNO_3_S	*Z* = 2
*M**_r_* = 261.72	*F*(000) = 272
Triclinic, *P*1	*D*_x_ = 1.389 Mg m^−^^3^
Hall symbol: -P 1	Mo *K*α radiation, λ = 0.71073 Å
*a* = 6.207 (1) Å	Cell parameters from 1240 reflections
*b* = 10.395 (3) Å	θ = 3.3–27.8°
*c* = 10.497 (3) Å	µ = 0.46 mm^−^^1^
α = 70.150 (2)°	*T* = 293 K
β = 79.160 (2)°	Plate, colourless
γ = 86.010 (2)°	0.46 × 0.20 × 0.08 mm
*V* = 625.7 (3) Å^3^	

### Data collection

**Table:**

Oxford Diffraction Xcalibur diffractometer with a Sapphire CCD detector	2476 independent reflections
Radiation source: fine-focus sealed tube	1842 reflections with *I* > 2σ(*I*)
graphite	*R*_int_ = 0.014
Rotation method data acquisition using ω and φ scans	θ_max_ = 26.4°, θ_min_ = 3.3°
Absorption correction: multi-scan *CrysAlis RED* (Oxford Diffraction, 2009)	*h* = −7→7
*T*_min_ = 0.815, *T*_max_ = 0.964	*k* = −12→12
3846 measured reflections	*l* = −13→13

### Refinement

**Table:**

Refinement on *F*^2^	Primary atom site location: structure-invariant direct methods
Least-squares matrix: full	Secondary atom site location: difference Fourier map
*R*[*F*^2^ > 2σ(*F*^2^)] = 0.060	Hydrogen site location: inferred from neighbouring sites
*wR*(*F*^2^) = 0.126	H atoms treated by a mixture of independent and constrained refinement
*S* = 1.19	*w* = 1/[σ^2^(*F*_o_^2^) + (0.0233*P*)^2^ + 0.632*P*] where *P* = (*F*_o_^2^ + 2*F*_c_^2^)/3
2476 reflections	(Δ/σ)_max_ = 0.001
148 parameters	Δρ_max_ = 0.28 e Å^−^^3^
1 restraint	Δρ_min_ = −0.31 e Å^−^^3^

### Special details

**Table:**

Geometry. All e.s.d.'s (except the e.s.d. in the dihedral angle between two l.s. planes) are estimated using the full covariance matrix. The cell e.s.d.'s are taken into account individually in the estimation of e.s.d.'s in distances, angles and torsion angles; correlations between e.s.d.'s in cell parameters are only used when they are defined by crystal symmetry. An approximate (isotropic) treatment of cell e.s.d.'s is used for estimating e.s.d.'s involving l.s. planes.
Refinement. Refinement of *F*^2^ against ALL reflections. The weighted *R*-factor *wR* and goodness of fit *S* are based on *F*^2^, conventional *R*-factors *R* are based on *F*, with *F* set to zero for negative *F*^2^. The threshold expression of *F*^2^ > σ(*F*^2^) is used only for calculating *R*-factors(gt) *etc*. and is not relevant to the choice of reflections for refinement. *R*-factors based on *F*^2^ are statistically about twice as large as those based on *F*, and *R*- factors based on ALL data will be even larger.

### Fractional atomic coordinates and isotropic or equivalent isotropic displacement parameters (Å^2^)

**Table:**

	*x*	*y*	*z*	*U*_iso_*/*U*_eq_	
C1	0.1905 (5)	0.2375 (3)	0.9043 (3)	0.0475 (7)	
C2	−0.0001 (6)	0.2717 (4)	0.8506 (4)	0.0610 (9)	
H2	−0.0818	0.3477	0.8593	0.073*	
C3	−0.0683 (6)	0.1930 (4)	0.7844 (4)	0.0670 (10)	
H3	−0.1957	0.2156	0.7468	0.080*	
C4	0.0535 (7)	0.0805 (3)	0.7742 (4)	0.0638 (10)	
C5	0.2423 (7)	0.0456 (4)	0.8278 (4)	0.0709 (11)	
H5	0.3221	−0.0315	0.8207	0.085*	
C6	0.3124 (6)	0.1255 (3)	0.8920 (4)	0.0612 (9)	
H6	0.4421	0.1040	0.9271	0.073*	
C7	0.4540 (6)	0.5278 (4)	0.7543 (4)	0.0578 (9)	
C8	0.4194 (7)	0.6715 (4)	0.6608 (4)	0.0716 (11)	
H8	0.3561	0.7263	0.7187	0.086*	
C9	0.2542 (9)	0.6652 (6)	0.5756 (5)	0.1190 (19)	
H9A	0.1202	0.6271	0.6350	0.143*	
H9B	0.3103	0.6089	0.5208	0.143*	
H9C	0.2265	0.7558	0.5165	0.143*	
C10	0.6291 (10)	0.7353 (6)	0.5780 (6)	0.151 (3)	
H10A	0.6959	0.6822	0.5219	0.182*	
H10B	0.7258	0.7386	0.6383	0.182*	
H10C	0.6015	0.8265	0.5202	0.182*	
N1	0.3069 (5)	0.4914 (3)	0.8767 (3)	0.0560 (7)	
H1N	0.200 (4)	0.541 (3)	0.892 (4)	0.067*	
O1	0.0960 (4)	0.3525 (3)	1.0924 (2)	0.0729 (7)	
O2	0.4794 (4)	0.2850 (3)	1.0314 (3)	0.0714 (7)	
O3	0.5885 (4)	0.4482 (3)	0.7273 (3)	0.0863 (9)	
Cl1	−0.0354 (3)	−0.01843 (12)	0.69033 (14)	0.1076 (5)	
S1	0.27563 (15)	0.33738 (9)	0.99054 (9)	0.0559 (3)	

### Atomic displacement parameters (Å^2^)

**Table:**

	*U*^11^	*U*^22^	*U*^33^	*U*^12^	*U*^13^	*U*^23^
C1	0.0524 (18)	0.0386 (16)	0.0429 (17)	0.0085 (14)	−0.0084 (14)	−0.0043 (13)
C2	0.055 (2)	0.056 (2)	0.074 (2)	0.0148 (16)	−0.0162 (18)	−0.0244 (18)
C3	0.063 (2)	0.063 (2)	0.072 (2)	0.0024 (18)	−0.0210 (19)	−0.014 (2)
C4	0.090 (3)	0.0428 (19)	0.053 (2)	−0.0118 (18)	−0.0140 (19)	−0.0066 (16)
C5	0.095 (3)	0.0419 (19)	0.077 (3)	0.0201 (19)	−0.026 (2)	−0.0180 (18)
C6	0.068 (2)	0.0471 (19)	0.070 (2)	0.0184 (17)	−0.0255 (19)	−0.0175 (17)
C7	0.052 (2)	0.059 (2)	0.059 (2)	0.0048 (17)	−0.0146 (17)	−0.0148 (17)
C8	0.086 (3)	0.062 (2)	0.057 (2)	0.007 (2)	−0.012 (2)	−0.0084 (18)
C9	0.146 (5)	0.114 (4)	0.095 (4)	0.039 (4)	−0.060 (4)	−0.020 (3)
C10	0.123 (5)	0.128 (5)	0.133 (5)	−0.026 (4)	−0.001 (4)	0.041 (4)
N1	0.0637 (19)	0.0447 (16)	0.0567 (17)	0.0105 (13)	−0.0102 (15)	−0.0155 (13)
O1	0.0929 (19)	0.0657 (16)	0.0489 (14)	0.0221 (14)	−0.0029 (13)	−0.0142 (12)
O2	0.0769 (17)	0.0681 (16)	0.0755 (17)	0.0171 (13)	−0.0381 (14)	−0.0221 (13)
O3	0.0719 (18)	0.087 (2)	0.0815 (19)	0.0296 (15)	−0.0026 (15)	−0.0156 (16)
Cl1	0.1677 (13)	0.0641 (7)	0.1053 (9)	−0.0180 (7)	−0.0502 (9)	−0.0294 (6)
S1	0.0676 (6)	0.0489 (5)	0.0494 (5)	0.0139 (4)	−0.0159 (4)	−0.0140 (4)

### Geometric parameters (Å, °)

**Table:**

C1—C6	1.374 (4)	C7—C8	1.510 (5)
C1—C2	1.378 (4)	C8—C10	1.481 (6)
C1—S1	1.753 (3)	C8—C9	1.500 (6)
C2—C3	1.370 (5)	C8—H8	0.9800
C2—H2	0.9300	C9—H9A	0.9600
C3—C4	1.372 (5)	C9—H9B	0.9600
C3—H3	0.9300	C9—H9C	0.9600
C4—C5	1.368 (5)	C10—H10A	0.9600
C4—Cl1	1.736 (4)	C10—H10B	0.9600
C5—C6	1.368 (5)	C10—H10C	0.9600
C5—H5	0.9300	N1—S1	1.639 (3)
C6—H6	0.9300	N1—H1N	0.840 (18)
C7—O3	1.199 (4)	O1—S1	1.433 (2)
C7—N1	1.380 (4)	O2—S1	1.425 (2)
			
C6—C1—C2	120.7 (3)	C10—C8—H8	107.9
C6—C1—S1	119.9 (3)	C9—C8—H8	107.9
C2—C1—S1	119.4 (2)	C7—C8—H8	107.9
C3—C2—C1	119.5 (3)	C8—C9—H9A	109.5
C3—C2—H2	120.2	C8—C9—H9B	109.5
C1—C2—H2	120.2	H9A—C9—H9B	109.5
C2—C3—C4	119.2 (3)	C8—C9—H9C	109.5
C2—C3—H3	120.4	H9A—C9—H9C	109.5
C4—C3—H3	120.4	H9B—C9—H9C	109.5
C5—C4—C3	121.5 (3)	C8—C10—H10A	109.5
C5—C4—Cl1	119.8 (3)	C8—C10—H10B	109.5
C3—C4—Cl1	118.7 (3)	H10A—C10—H10B	109.5
C6—C5—C4	119.2 (3)	C8—C10—H10C	109.5
C6—C5—H5	120.4	H10A—C10—H10C	109.5
C4—C5—H5	120.4	H10B—C10—H10C	109.5
C5—C6—C1	119.8 (3)	C7—N1—S1	125.8 (2)
C5—C6—H6	120.1	C7—N1—H1N	122 (3)
C1—C6—H6	120.1	S1—N1—H1N	110 (3)
O3—C7—N1	121.2 (3)	O2—S1—O1	119.07 (16)
O3—C7—C8	125.4 (3)	O2—S1—N1	110.56 (16)
N1—C7—C8	113.3 (3)	O1—S1—N1	103.75 (15)
C10—C8—C9	113.5 (4)	O2—S1—C1	108.79 (15)
C10—C8—C7	111.6 (4)	O1—S1—C1	108.78 (17)
C9—C8—C7	107.7 (4)	N1—S1—C1	104.95 (15)
			
C6—C1—C2—C3	0.0 (5)	N1—C7—C8—C9	−87.3 (4)
S1—C1—C2—C3	−179.3 (3)	O3—C7—N1—S1	−7.0 (5)
C1—C2—C3—C4	0.7 (6)	C8—C7—N1—S1	170.6 (3)
C2—C3—C4—C5	−0.5 (6)	C7—N1—S1—O2	54.8 (3)
C2—C3—C4—Cl1	179.9 (3)	C7—N1—S1—O1	−176.4 (3)
C3—C4—C5—C6	−0.6 (6)	C7—N1—S1—C1	−62.3 (3)
Cl1—C4—C5—C6	179.1 (3)	C6—C1—S1—O2	2.9 (3)
C4—C5—C6—C1	1.4 (6)	C2—C1—S1—O2	−177.8 (3)
C2—C1—C6—C5	−1.1 (5)	C6—C1—S1—O1	−128.3 (3)
S1—C1—C6—C5	178.3 (3)	C2—C1—S1—O1	51.1 (3)
O3—C7—C8—C10	−35.0 (6)	C6—C1—S1—N1	121.2 (3)
N1—C7—C8—C10	147.5 (4)	C2—C1—S1—N1	−59.4 (3)
O3—C7—C8—C9	90.2 (5)		

### Hydrogen-bond geometry (Å, °)

**Table:**

*D*—H···*A*	*D*—H	H···*A*	*D*···*A*	*D*—H···*A*
N1—H1N···O1^i^	0.84 (2)	2.08 (2)	2.912 (4)	169 (3)

Symmetry codes: (i) −*x*, −*y*+1, −*z*+2.
